# Efficacy and Safety of Artesunate + Amodiaquine and Artemether + Lumefantrine for the Treatment of Uncomplicated *Plasmodium falciparum* Malaria in Madagascar, 2020

**DOI:** 10.4269/ajtmh.24-0844

**Published:** 2025-11-04

**Authors:** Aina Harimanana, Dina Ny Aina Liantsoa Randriamiarinjatovo, Tovonanahary Angelo Rakotomanga, Judickaelle Irinantenaina, Leah F. Moriarty, Veronica Laird, Dhruviben Patel, Rispah A. Abdallah, Zhou Zhiyong, Samaly Souza Svigel, Laurent Kapesa, Jocelyn Razafindrakoto, Fanomezantsoa Ralinoro, Marie Ange Rason, Mauricette Nambinisoa Andriamananjara, Seheno Razanatsiorimalala, Celestin Razafinjato, Milijaona Randrianarivelojosia

**Affiliations:** ^1^ Institut Pasteur de Madagascar, Antananarivo, Madagascar;; ^2^ Programme National de Lutte Contre le Paludisme, Ministère de la Santé Publique de Madagascar, Antananarivo, Madagascar;; ^3^ US President’s Malaria Initiative, Malaria Branch, Division of Parasitic Diseases and Malaria, Centers for Disease Control and Prevention, Atlanta, Georgia;; ^4^ Oak Ridge Institute for Science and Education, Oak Ridge, Tennessee;; ^5^ Division of Parasitic Diseases and Malaria, Centers for Disease Control and Prevention, Atlanta, Georgia;; ^6^ US President’s Malaria Initiative, US Agency for International Development, Antananarivo, Madagascar;; ^7^ PMI EVOLVE Project, Antananarivo, Madagascar;; ^8^ Université de Toliara, Toliara, Madagascar

## Abstract

The efficacy of artesunate + amodiaquine (ASAQ) and artemether + lumefantrine (AL) for treating malaria was investigated in Madagascar in 2020. A randomized parallel-group study was conducted at four health centers (Antsenavolo, Vohitromby, and Matanga in the southeastern region and Ankazomborona in the northwestern region). The therapeutic efficacy and safety of ASAQ and AL were assessed using the WHO protocol, with a 28-day follow-up period. Children aged 6 months to 14 years with uncomplicated *Plasmodium falciparum* (*P. falciparum*) malaria were randomly assigned to receive either ASAQ or AL. Genotyping assays for the *pfK13* gene were conducted on *P. falciparum* isolates obtained from dry blood samples collected on Day 0. Of 765 enrolled patients, 709 (92.7%) reached the study endpoint. Among the per-protocol population, crude adequate clinical and parasitological response (ACPR) rates were 99%, 99%, 100%, and 100% for Antsenavolo, Vohitromby, Matanga, and Ankazomborona, respectively, in the ASAQ group and 98%, 91%, 90%, and 100% for the same locations, respectively, in the AL group. Polymerase chain reaction-corrected ACPR rates were 100% for the ASAQ group at all study sites, whereas for the AL group, they were 98.8% in Antsenavolo, 97.6% in Vohitromby, 100% in Matanga, and 100% in Ankazomborona. Day 3 slide positivity rates were 0%, 1%, 1%, and 0% for Antsenavolo, Vohitromby, Matanga, and Ankazomborona, respectively. During follow-up, mild and moderate adverse events, including gastrointestinal issues (abdominal pain and vomiting) and headache, were reported in 10.2% (72/709) of patients. Of 727 samples successfully analyzed for *pfK13*, no mutation associated with artemisinin resistance was observed. The study results reveal that ASAQ and AL remain safe and efficacious for treating uncomplicated *P. falciparum* malaria in Madagascar.

## INTRODUCTION

On the island of Madagascar, malaria remains a major public health problem. Malaria is perennial in coastal areas, and *Plasmodium falciparum* (*P. falciparum*) is the predominant malaria species. Effective case management, consisting of early diagnosis, prompt treatment, and prevention with antimalarial drugs, is a key component in achieving malaria elimination. Since 2005, the national malaria policy has adopted artemisinin-based combination therapies (ACTs) and recommended the combinations of artesunate + amodiaquine (ASAQ) and artemether + lumefantrine (AL) for treating uncomplicated malaria. Over the last decade, previous studies have revealed the efficacy of these drugs against *P. falciparum* malaria.^1^^–^^4^

However, the emergence of antimalarial resistance in Asia,^5^^,^^6^ as well as the emergence of artemisinin partial resistance in Africa, remains a threat to malaria control.^7^^–^^9^ It is therefore crucial to monitor the efficacy of these ACTs. In line with the WHO’s recommendations, antimalarial drug resistance was monitored in Madagascar to ensure that the Ministry of Public Health is up-to-date on the efficacy of antimalarial drugs. Therefore, a therapeutic efficacy study was conducted to assess the efficacy of ASAQ and AL in the southeastern and northwestern coastal areas of Madagascar.

## MATERIALS AND METHODS

The present work was a multisite, randomized, controlled study conducted to assess the efficacy and tolerability of ASAQ and AL combinations for treating uncomplicated *P. falciparum* malaria in children using the WHO protocol for the surveillance of anti-malarial drug efficacy.^10^

### Study sites.

**Figure 1.**
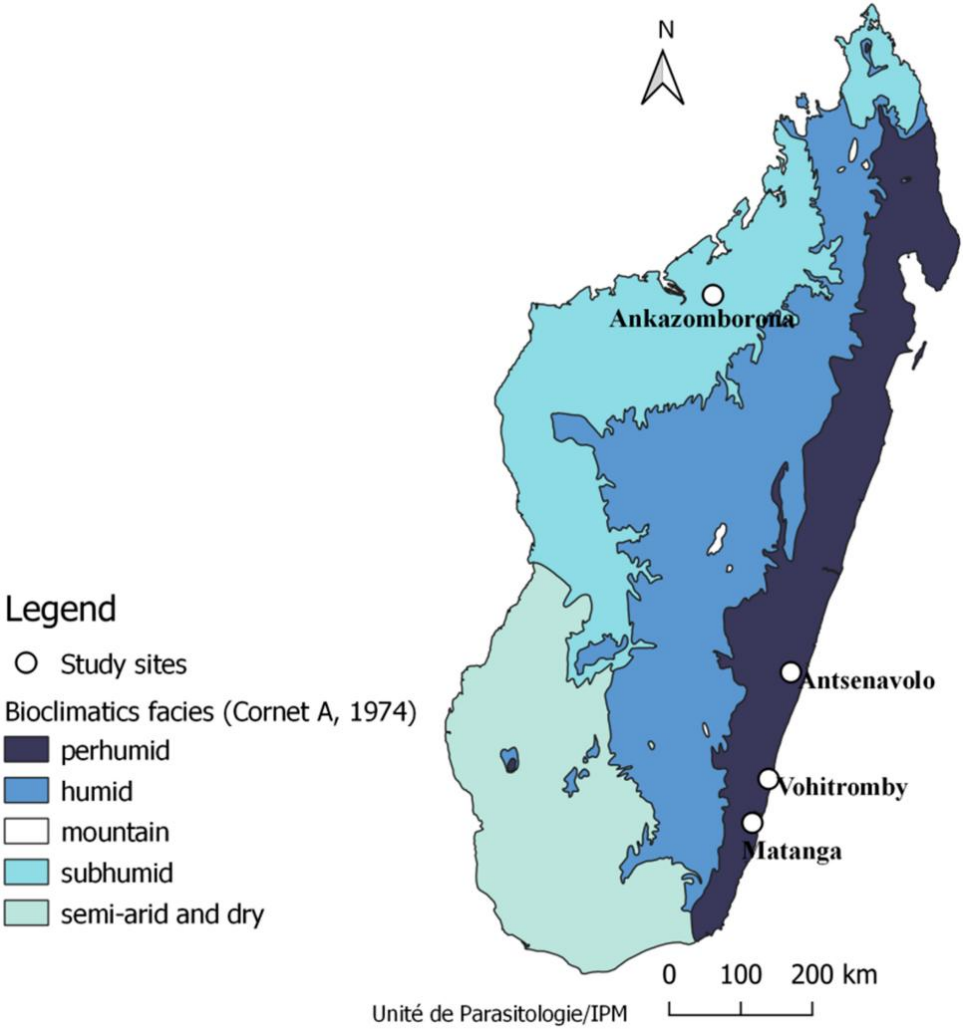
Location of TES sites, 2020.

The current study was conducted at four sites: one site in the northwestern coastal area (Ankazomborona in the health district of Marovoay) and three sites in the southeastern coastal area (Antsenavolo, in the health district of Mananjary; Vohitromby, in the health district of Farafangana; and Matanga, in the health district of Vangaindrano; Figure 1). Malaria transmission is perennial at these sites. According to the National Malaria Control Program in Madagascar, the annual incidence of malaria in these study sites exceeds 100‰. The southeastern study sites have high annual rainfall (2,500 mm), with average annual temperatures ranging from 15°C to 32°C.^11^^–^^13^ In contrast, the northwestern study site has less annual rainfall (1,500 mm) and an average annual temperature of 29°C.^14^

### Patient screening.

Patients were enrolled between March and October 2020. Children aged between 6 months and 14 years who had a fever or a history of fever in the previous 24 hours seeking treatment at the study sites were examined by nurses and tested for malaria using a rapid diagnostic test (mRDT). The SD Bioline Malaria Ag P.f/*Pan* test (Standard Diagnostics, Inc., Suwon, South Korea, Batch 05EDD019A) was used in the present study. Children with a positive mRDT result and no other illness who required immediate attention were referred to the study team for screening procedures. In accordance with the WHO protocol, the study included patients infected with *P. falciparum* who resided in the study areas throughout the planned period met the following criteria: aged between 6 months and 14 years old; weighing ≥5 kg; displaying parasitemia ranging from 1,000 to 100,000 asexual forms per microliter of blood; a hemoglobin level ≥8 g/dl; without signs of severe malnutrition; presenting with an axillary temperature ≥37.5°C or reporting fever within the last 24 hours; and able to tolerate oral treatment.^10^

Patients with any clinical sign of severe malaria^15^ or any serious concomitant disease, such as cardiovascular disease, were excluded. Pregnant or breastfeeding women were also excluded, and a urine pregnancy test was performed on all women of childbearing age. Preliminary results from the demographic and health surveys revealed early fertility at 10 years old. A pregnancy test was performed for all women over 10 years of age. Successive enrollments in the present study or simultaneous enrollment in any other clinical trial were also prohibited.

### Randomization and treatment.

Patients at each study site were randomly assigned to one of two treatment arms on Day 0: ASAQ (Ipca Laboratories, Ltd., Mumbai, India, and Winthrop SANOFI, Casablanca, Morocco), administered once daily, and AL (Coartem® NOVARTIS, Basel, Switzerland), administered twice daily. Randomization was stratified according to patient age using separate computer-generated randomization lists. Three age strata were used: 6 to 59 months, 5 to 9 years, and 10 to 14 years. Treatment information was placed in a sealed envelope. Each study participant was randomly assigned (1:1) to receive either ASAQ or AL. All doses of these drugs were administered and directly observed by the study’s medical teams.

Treatment dosages were determined on the basis of each patient’s body weight in accordance with national treatment guidelines.^16^ Treatment duration was 3 days. The ASAQ treatment was administered once a day, with an interval of 24 hours. The following weight bands were used for dosing children: 5 to <9 kg, one tablet containing 25 mg of artesunate (AS) and 67.5 mg of amodiaquine (AQ); 9 to <18 kg, one tablet containing 50 mg of AS and 67.5 mg of AQ; 18 to <36 Kg, one tablet containing 100 mg of AS and 270 mg of AQ; >36 kg, two tablets, each containing 100 mg of AS and 270 mg of AQ. The AL treatment was administered twice daily, with an 8-hour interval between the first and second doses on Day 0 and a 12-hour interval between the two daily doses on Days 1 and 2. The AL tablet contained 20 mg of artemether and 120 mg of lumefantrine: for children weighing 5 to <15 kg, one tablet/dose; children weighing 15 to <25 kg received two tablets per dose; children weighing 25 to <35 kg received three tablets per dose; and children weighing ≥35 kg received four tablets per dose. Tablets were administered orally with a small amount of water in the presence of study medical staff members, and patients were advised to resume a normal diet as soon as possible. Before drug administration, all children were given cookies. All patients were monitored for 30 minutes after drug administration to ensure that the drug was not vomited. If vomiting occurred, the dose was readministered. If vomiting occurred a second time, the subject was withdrawn from the study and referred to the health facility for treatment (parenteral artesunate). The study patients did not need to be moved to take their treatment dose; they stayed at the study center for the 3 days of treatment.

### Efficacy and safety of treatment.

The WHO protocol with a 28-day follow-up period was used to assess the therapeutic efficacy of the antimalarial drugs.^10^ Follow-up visits were systematically performed on Days 1, 2, 3, 7, 14, 21, and 28 after enrollment (Table 1). Each visit consisted of a history and physical examination, which combined an evaluation of clinical safety with the measurement of vital signs. Axillary temperature was measured using an infrared thermometer. Blood pressure and pulse were measured after a 10-minute rest in a sitting position. Finger-prick blood samples were collected for malaria blood smears and dried blood spots, which would be used for parasite count and polymerase chain reaction (PCR) analysis. On Days 0 and 28, hemoglobin concentration was measured.

**Table 1:** Follow-up schedule

Study Parameters	Reference Days								Unplanned Visit
	D0	D1	D2	D3	D7 ±1day	D14 ±1day	D21 ±1day	D28 ±1day	
mRDT	x	–	–	–	–	–	–	–	–
Clinical examination	x	x	x	x	x	x	x	x	–
Hemoglobin	x	–	–	–	–	–	–	x	–
Physical examination/vital signs	x	x	x	x	x	x	x	x	x
Parasitological examination	x	x	x	x	x	x	x	x	x
Preparation of filter paper blood spot samples for PCR analysis	x	x	x	x	x	x	x	x	x

D = day; mRDT = malaria rapid diagnostic test; PCR = polymerase chain reaction.

Clinical efficacy was assessed by grading clinical signs and measuring temperature. Parasitological clearance was based on asexual parasitemia, although a gametocyte count was also systematically performed. The following clinical symptoms were assessed systematically at each visit and graded as absent, mild, moderate, severe, or very severe: diaphoresis, headache, chills, pain (specifying location), jaundice, asthenia, dizziness, anorexia, reduced skin turgor, skin rash, and pruritus. The severity grading scales were adapted from guidelines provided by the WHO (Toxicity Grading Scale for Determining the Severity of Adverse Event).^10^^,^^17^ Reports of vomiting and diarrhea were noted. The primary endpoint was the rate of adequate parasitological and clinical response after PCR correction on Day 28, in accordance with the 2009 in vivo WHO protocol. Clinical safety was monitored through regular patient interviews regarding adverse events that occurred after the previous visit.

### Study withdrawal.

The patients, or their parents or guardians, were free to withdraw from the study or stop taking treatment at any point, irrespective of the reason; they were also free to withdraw on the basis of the investigator’s decision in case of onset of any danger signs of severe malaria or any adverse event justifying treatment discontinuation. Study withdrawal was documented by the investigator on the case report form (CRF). When possible, patients were evaluated according to end-of-study visit procedures. Patients presenting with severe disease or adverse events were referred to the nearest hospital for medical care.

Before each patient’s visit, the study team made a phone call to remind the patient about the upcoming visit date when possible. At each visit, patients were given a travel expense allowance. In cases in which a patient missed a visit, the study team made three follow-up visits to the patient’s home. If the patients were not available after three attempts, they were declared “lost to follow-up.” For patients lost to follow-up, the CRF was completed up to the most recent visit. No patients who had been prematurely withdrawn from the study were replaced.

### Laboratory analysis.

During enrollment and at subsequent follow-up visits, thin and thick blood smears were obtained and stained using May-Grünwald Giemsa. Two sets of thick and thin blood films were prepared using 3 *µ*L or 6 *µ*L of whole blood at the time of enrolment and during each scheduled and unscheduled visit. To create dried blood spots (DBSs), 200 microliters of whole blood was deposited on filter paper cards (DBS Whatman® 903 protein saver cards, batch number: 7078317W162, GE Healthcare Ltd., Cardiff, United Kingdom), dried overnight, and stored at room temperature with individual desiccant packs. Giemsa-stained thick blood smears were examined onsite by the microscopy practitioners. Each site had a total of three qualified microscopists. Two qualified microscopists examined all slides independently, and parasite densities were calculated by averaging the two counts. Blood smears with discordant results (differing results obtained by the two microscopists regarding species diagnosis, parasite density ≥25%, or the detection and identification of parasites) were re-examined by a third independent microscopist, and parasite density was calculated by averaging the two closest counts.^10^ Parasite densities were calculated by counting the number of asexual and sexual *P. falciparum* parasites until 500 leukocytes were observed and then converting this figure into the number of parasites (trophozoites and/or gametocytes) per microliter of blood, assuming an average leukocyte count of 8,000/*µ*L. Microscopy results were considered negative if no parasites were seen after 1,000 leukocytes had been observed. The presence of gametocytes on an enrollment or follow-up slide has been noted, but this information did not contribute to the basic evaluation. Hemoglobin was measured in capillary blood using a portable hemoglobin analyzer HemoCue Hb 201 System (HemoCue AB, Angelholm, Sweden).

### Polymerase chain reaction analysis.

#### Microsatellite genotyping.

All dried blood spots were transported to the Institut Pasteur de Madagascar (for Antsenavolo and Vohitromby) and the National Malaria Control Program (for Ankazomborona and Matanga) laboratories in Antananarivo. For participants with recurrent parasitemia, DBSs collected on Day 0 and the day of parasitemia recurrence (day of failure [DoF]) were analyzed via PCR to allow neutral microsatellites to distinguish between reinfection and recrudescence.^3^^,^^10^^,^^18^ Parasite DNA was obtained from Day 0 and DoF samples. Dried blood spots from late therapeutic failure specimens (Day 21 and Day 28) were shipped to the CDC malaria laboratory in Atlanta, GA. Four 3 mm punches from a DBS sample were used to extract genomic DNA using the QIAamp® DNA Mini Kit (QIAGEN, Valencia, CA), according to the manufacturer’s instructions. The DNA was eluted in 200 *µ*L of elution buffer, aliquoted, and stored at –20°C until use. The presence of *P. falciparum* DNA was confirmed via photo-induced electron transfer PCR.^19^ Seven neutral microsatellites were amplified from MSTA1 (chromosome 6), MSPoly-α (chromosome 4), MSPfPK2 (chromosome 12), MSTA109 (chromosome 6), MS2490 (chromosome 10), MSC2M34 (chromosome 2), and MSC3M69 (chromosome 3) using PCR.^20^ Subsequently, fragment electrophoresis was performed on a capillary sequencer using an ABI 3,130 xl Genetic Analyzer (Applied Biosystems, Waltham, MA). GeneMarker software was used for fragment analysis. Day 0 and DoF alleles were compared. A previously described Bayesian algorithm^21^ was used to distinguish between reinfection and recrudescence.

#### pfk13 genotyping.

Blood spots collected on Day 0 and during follow-up visits after Day 3 were dried and stored in individual Ziploc bags with desiccant. All baseline Day 0 samples and DoF samples from participants with recurrent parasitemia were included in the molecular analysis at the US CDC. A total of 727 samples were included, including 70 samples that were processed individually and 657 D0 non-failure samples that were pooled before DNA extraction. A total of 171 pools were constructed from the 657 D0 non-failure samples. For each study site, the D0 non-failure samples were aligned from lowest to highest parasite density, as measured via microscopy, and sorted into pools. Polymerase chain reaction tests were performed to amplify the full-length *pfk13* genes from 70 individual patient samples and 171 pools using a previously described protocol.^22^ The amplification products were sequenced using the Malaria Resistance Surveillance protocol.^23^ In brief, unique sequence indices were added to PCR amplicons for the 70 individual patient samples and the 171 pools using the Illumina Library kit (Illumina, Inc., San Diego, CA). One sequencing run was performed. Sequences were analyzed at a locus representing the major reportable single-nucleotide polymorphisms. A sample was considered a sequencing success if a percent quality of 30 or higher and more than five reads were observed at the locus. Single-nucleotide polymorphisms were analyzed using a standardized workflow in Geneious Prime (Biomatters, San Francisco, CA).

### Sample size calculation.

The number of subjects was calculated on the basis of 28-day PCR-corrected efficacy. The success rate hypothesis (95%), a 95% CI with a type I (α) error of 5% and a type II (β) error of 20%, and a risk of patients lost to follow-up of 15%, were taken into account. A minimum of 86 patients per arm was required for each study site.

## STATISTICAL ANALYSES

The primary endpoint was the 28-day uncorrected and PCR-corrected treatment efficacy. Treatment outcomes were assessed according to the WHO guidelines:^10^ early treatment failures (ETFs), including danger signs, severe malaria, or failure to adequately respond to therapy from Day 0 to Day 3; late clinical failures (LCFs), characterized by danger signs, complicated malaria, or fever and parasitemia from Day 4 to Day 28 without previously meeting criteria for ETF; late parasitological failure (LPF), marked by asymptomatic parasitemia from Day 4 to Day 28 without previously meeting the criteria for ETF or LCF; and adequate clinical and parasitological response (ACPR), indicated by the absence of parasitemia on Day 28 without previously meeting the criteria for ETF, LCF, or LPF.^10^ Secondary endpoints included parasitemia, gametocyte carriage, and hemoglobin level.

Descriptive analysis was conducted using frequencies and percentages for qualitative variables and medians and interquartile intervals (IIQs) for quantitative variables. Categorical variables were compared using the χ^2^ test or Fisher’s exact test, whereas quantitative variables were analyzed using the Wilcoxon signed-rank test. The uncorrected and PCR-corrected per-protocol efficacy for each site and drug was calculated by dividing the number of participants classified as ACPR by the number of all participants who reached a study outcome. The sum of posterior probabilities of recrudescence was used to calculate the total number of recrudescent infections in the PCR-corrected analysis.

Reinfections were removed from the calculations of PCR-corrected per-protocol efficacy. For Kaplan–Meier cumulative efficacy estimates, reinfections were removed (censored) on the day of reinfection, and participants who were lost to follow-up or withdrew were included until the last day of follow-up in both uncorrected and PCR-corrected analyses. Posterior sampling was used to generate the PCR-corrected Kaplan–Meier estimates and 95% CIs using the posterior probabilities of recrudescence.

Data were recorded using the WHO-standard template. The Open Data Kit system (ODK, San Diego, CA) was used on an Android tablet to send collected data to the database in real time. Data cleaning was performed regularly to identify and correct errors. Statistical analysis was performed using STATA 15 (StatCorp LLC, College Station, TX).

## RESULTS

**Figure 2.**
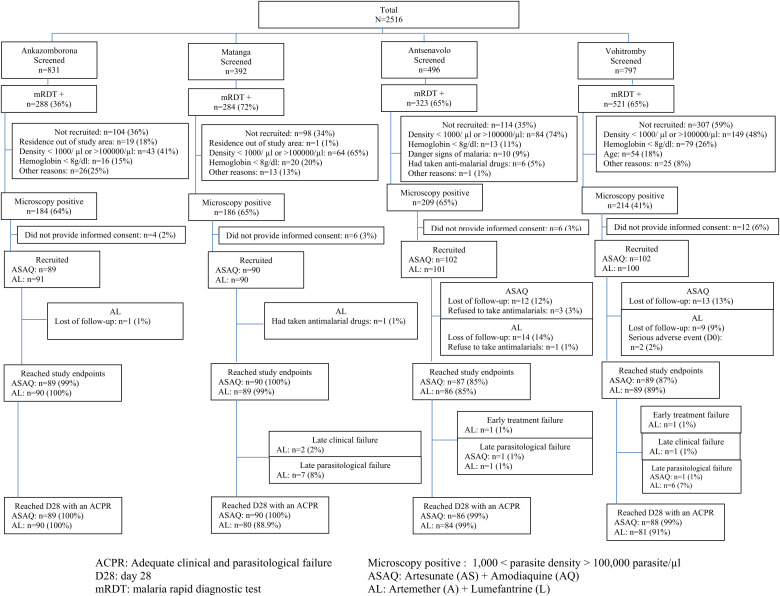
Participant dispositions and antimalarial therapeutic efficacy, Madagascar 2020.

Overall, 765 patients enrolled from four study sites were randomly assigned to one of the two therapeutic arms. The flow of patients through the study is displayed in Figure 2. Patient features at enrollment are presented in Table 2. A total of 56 patients (7.3%) were excluded because of interrupted follow-up (*n* = 48), which occurred after the withdrawal of the field medical team due to the spread of coronavirus disease 2019 in southeastern Madagascar, as declared by the Ministry of Public Health. Exclusion also occurred because of children’s refusal to swallow anti-malarial drugs (*n* = 4), serious adverse events (*n* = 2), loss to follow-up (*n* = 1), and patients having taken anti-malarial drugs before enrollment (*n* = 1). On Day 28, the per-protocol population consisted of 709 patients.

**Table 2:** Number of participants screened, enrolled, and finishing follow-up, as well as characteristics at baseline, collected as part of therapeutic efficacy monitoring, Madagascar 2020

Parameter	Ankazomborona		Matanga		Antsenavolo		Vohitromby	
	ASAQ	AL	ASAQ	AL	ASAQ	AL	ASAQ	AL
Number of participants screened	831	–	392	–	496	–	797	–
Number enrolled	89	91	90	90	102	101	102	100
Number lost to follow-up (%)	–	1 (1)	–	–	12 (12)	14 (14)	13 (13)	9 (9)
Number excluded (%)	–	–	–	1 (1)	3 (3)	1 (1)	–	2 (2)
Number that reached study endpoint (%)	89 (100)	90 (99)	90 (100)	89 (99)	87 (85)	86 (85)	89 (87)	89 (89)
Participant characteristics at baseline								
Median age, months (range)	8 (5–12)	7 (5–11)	7 (4–10)	7 (5–10)	6 (5–11)	7 (4–10)	8 (5–10)	7 (5–10)
Median weight, kg (range)	20 (12–35)	22 (15–27)	20 (14–26)	19 (16–26)	19 (13–25)	18 (13–25)	19 (15–23)	19 (14–24)
% of female participants	40	48	50	56	41	50	50	50
Median Day 0 parasitemia, number of parasites/*µ*L (range)	36,686 (13,798–67,435)	28,650 (13,891–60,296)	21,014 (7,800–38,651)	26,204 (6,489–51,566)	11,047 (4,156–29,755)	8,371 (3,063–23,976)	13,405 (4,529–28,879)	14,518 (7,028–32,632)
Median Day 0 hemoglobin level, g/dL (range)	10.3 (9.2–11.4)	10.5 (9.2–11.9)	11.4 (10–12.5)	11.6 (10.5–12.4)	10 (9.1–11.1)	10.2 (9.2–11)	10.4 (9.1–11.5)	10.8 (9.6–11.8)
Start month to end month	May–Aug		May–Aug		Mar–Sept		Mar–Oct	

AL = artemether + lumefantrine; ASAQ = artesunate + amodiaquine.

### Clinical and parasitological responses to treatments.

In the AL arm, on Day 1, two cases of early treatment failure were identified. Seventeen cases of late treatment failures were identified: five on Day 21 and 12 on Day 28. Most of these treatment failures were determined to be reinfections; one patient was classified as recrudescence on Day 28 (Table 3). Polymerase chain reaction-corrected and uncorrected clinical and parasitological cure rates on Day 28, calculated using the Kaplan–Meier estimate of the survival curve, are shown in Table 4. The crude cumulative efficacy in the AL arm was 100% (95% CI: 95.9–100) in Ankazomborona, 97.7% (95% CI: 91.0–99.4) in Antsenavolo, 91.1% (95% CI: 82.8–95.4) in Vohitromby, and 89.9% (95% CI: 81.5–94.6) in Matanga. After excluding reinfections, the corrected 28-day efficacy of AL was 100% (95% CI: 95.9–100) in Ankazomborona, 98.9% (95% CI: 91.9–99.8) in Antsenavolo, 97.6% (95% CI: 90.7–99.4) in Vohitromby, and 100% (95% CI: 95.5–100) in Matanga. Overall, the efficacy of AL was estimated to be 99.1% (95% CI: 97.2–99.7).

**Table 3:** Treatment outcomes for participants who completed follow-up as part of therapeutic efficacy monitoring, Madagascar 2020

	Number of Participants (%)							
	Ankazomborona		Matanga		Antsenavolo		Vohitromby	
Outcomes	ASAQ (*n* = 89)	AL (*n* = 90)	ASAQ (*n* = 90)	AL (*n* = 89)	ASAQ (*n* = 87)	AL (*n* = 86)	ASAQ (*n* = 89)	AL (*n* = 89)
Treatment failure	–	–	–	9 (10)	1 (1)	2 (2)	1 (1)	8 (9)
Early treatment failure	–	–	–	–	–	1 (1)	–	1 (1)
Recurrent parasitemia	–	–	–	9 (10)	1 (1)	1 (1)	1 (1)	7 (8)
Recrudescence	–	–	–	–	–	–	–	1 (1)
Day 14	–	–	–	–	–	–	–	–
Day 21	–	–	–	–	–	–	–	–
Day 28	–	–	–	–	–	–	–	1(1)
Reinfection	–	–	–	9 (10)	1 (1)	1 (1)	1 (1)	6 (7)
Day 14	–	–	–	–	–	–	–	–
Day 21	–	–	–	4 (5)	–	–	–	1 (1)
Day 28	–	–	–	5 (6)	1 (1)	1 (1)	1 (1)	5 (6)
Adequate clinical and parasitological response	89 (100)	90 (100)	90 (100)	80 (90)	86 (99)	84 (98)	88 (99)	81 (91)

AL = artemether + lumefantrine; ASAQ = artesunate + amodiaquine.

**Table 4:** Efficacy of first-line antimalarial drugs on day 28, Madagascar 2020

	Ankazomborona				Matanga				Antsenavolo				Vohitromby			
	ASAQ		AL		ASAQ		AL		ASAQ		AL		ASAQ		AL	
	%	95% CI	%	95% CI	%	95% CI	%	95% CI	%	95% CI	%	95% CI	%	95% CI	%	95% CI
Uncorrected PCR																
Per-protocol	100	95.9–100	100	95.9–100	100	96.0–100	89.9	81.5–94.6	98.9	92.1–99.8	97.7	91.0–99.4	98.8	96.7–100	91.1	82.8–95.4
Kaplan–Meier	100	95.9–100	100	96.0–100	100	96.0–100	89.9	81.5–94.6	98.9	92.1–99.8	97.7	91.0–99.4	98.8	96.7–100	91.0	82.3–95.4
Corrected PCR																
Per-protocol	100	95.9–100	100	95.9–100	100	96.0–100	100	95.5–100	100	95.8–100	98.8	91.9–99.8	100	95.9–100	97.6	90.7–99.4
Kaplan–Meier	100	95.9–100	100	95.9–100	100	96.0–100	100	65.5–100	100	95.8–100	98.8	92.0–99.8	100	95.9–100	97.7	91.2–99.4

AL = artemether + lumefantrine; ASAQ = artesunate + amodiaquine; PCR = polymerase chain reaction.

In the ASAQ arm, the uncorrected cumulative efficacy was 100% (95% CI: 95.9–100) in Ankazomborona, 98.9% (95% CI: 92.1–99.4) in Antsenavolo, 98.8% (95% CI: 96.7–100) in Vohitromby, and 100% (95% CI: 96.0–100) in Matanga. There were two cases of late treatment failure on Day 28. All treatment failures were classified as reinfections. After excluding reinfections, the corrected 28-day efficacy in the ASAQ arm was 100% (95% CI: 95.9–100) in Ankazomborona, 100% (95% CI: 95.8–100) in Antsenavolo, 100% (95% CI: 95.9–100) in Vohitromby, and 100% (95% CI: 96.0–100) in Matanga (Table 4). Overall, the efficacy of ASAQ was estimated to be 100% (95% CI: 99–100). Adjusting the therapeutic response rate by region and age did not reveal any significant differences.

### Parasitic clearance and resolution of clinical symptoms in the four sites.

**Figure 3.**
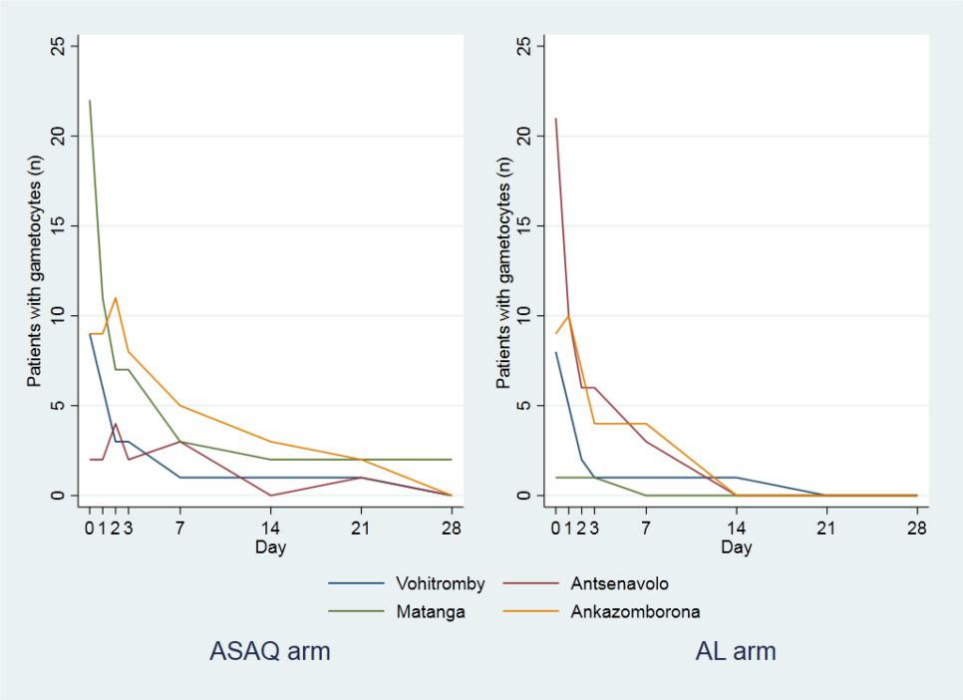
Gametocyte clearance by arm and study site and antimalarial therapeutic efficacy, Madagascar 2020.

At enrollment, in the AL arm, the median (IIQ) parasite densities varied from 8,371 parasites/*µ*L of blood (3,063–23,976) in Antsenavolo to 28,650 parasites/*µ*L of blood (13,891–60,296) in Ankazomborona; in the ASAQ arm, the median (IIQ) parasite densities varied from 11,047 parasites/*µ*L of blood (4,156–29,755) in Antsenavolo to 36,686 parasites/*µ*L of blood (13,798–67,435) in Ankazomborona. Four patients remained parasitemic on Day 3: 0.8% (3/356) in the ASAQ arm and 0.3% (1/354) in the AL arm. Their parasite densities ranged from 23 to 141 parasites/*µ*L of blood (Table 5), and they received no additional antimalarial treatment. On Day 7, all of them were aparasitemic. The number of gametocyte carriers decreased from Day 1 and reached zero on Day 21 in the AL arm at all study sites and on Day 28 in the ASAQ arm at three study sites, apart from Ankazomborona (Figure 3). The negativity rate on Day 2 ranged from 67% to <98%, whereas the negativity rate on Day 3 was greater than 99% for all study sites and arms (Table 6).

**Table 5:** Parasitic clearance, Madagascar 2020

	Ankazomborona		Matanga		Antsenavolo		Vohitromby	
	ASAQ, *N* = 89	AL, *N* = 90	ASAQ, *N* = 90	AL, *N* = 89	ASAQ, *N* = 87	AL, *N* = 86	ASAQ, *N* = 90	AL, *N* = 89
Day 1								
*N* (%)*	85 (95.5)	89 (98.9)	71 (78.9)	78 (87.6)	24 (27.6)	42 (48.8)	23 (25.6)	40 (44.9)
Density, median (IIQ)	484 (240–1,520)	545 (187–3,677)	452 (143–1,334)	792 (284–1,973)	117 (41–294)	205 (87–572)	122 (47–253)	135 (63–664)
Day 2								
*N* (%)*	25 (28.1)	30 (33.3)	7 (7.8)	12 (13.5)	2 (2.3)	2 (2.3)	4 (4.5)	3 (3.4)
Density, median (IIQ)	90 (66–203)	159 (87–250)	133 (103–770)	189 (102–237)	59 (40–77)	94 (47–141)	126 (64–172)	31 (16–63)
Day 3								
*N* (%)*	0	0	2 (2.2)	0	0	0	1 (1.1)	1 (1.1)
Density, median (IIQ)	–	–	141 (86–195)	–	–	–	31 (31–31)	23 (23–23)

AL = artemether + lumefantrine; ASAQ = artesunate + amodiaquine; IIQ = interquartile interval.

* Parasite carriage.

**Table 6:** Proportions of slides negative for asexual malaria parasites on Days 2 and 3 after antimalarial treatment administered during treatment efficacy monitoring, Madagascar 2020

Day	Ankazomborona				Matanga				Antsenavolo				Vohitromby			
	ASAQ		AL		ASAQ		AL		ASAQ		AL		ASAQ		AL	
	*n* = 89	%, 95% CI	*n* = 90	%, 95% CI	*n* = 90	%, 95% CI	*n* = 89	%, 95% CI	*n* = 87	%, 95% CI	*n* = 86	%, 95% CI	*n* = 90	%, 95% CI	*n* = 89	%, 95% CI
2	64	72	60	67	83	92	77	87	85	98	84	98	86	95	86	97
		62–80		56–76		85–96		78–92		91–99		91–99		88–98		90–99
3	89	100	90	100	88	98	89	100	87	100	86	100	89	99	88	99
		96–100		96–100		92–99.4		96–100		96–100		96–100		92–99		92–99

% = proportion; AL = artemether + lumefantrine; ASAQ = artesunate + amodiaquine.

### Drug safety.

Overall, 10.2% (72/709) of patients who reached a study endpoint presented with at least one clinical sign or symptom on Days 2 and 3. Among these symptoms, the most frequently reported included abdominal pain (4.5% [32/709]), anorexia (6.2% [44/709]), headache (0.9% [6/709]), cough (2.0% [14/709]), and vomiting (0.1% [1/709]). A significant difference between treatment arms was observed for abdominal pain (ASAQ: 7.3% [26/355] versus AL: 1.7% [6/354]; *P*-value <0.001) and anorexia (ASAQ: 9.9% [35/355] versus AL: 2.5% [9/354]; *P*-value <0.001). None of the adverse events were considered to be treatment-related by the investigators.

Hemoglobin values on Days 0 and 28 were available for 674 individuals. The median hemoglobin values exhibited a significant increase (*P*-value <0.001) from the initial value of 10.5 (IIQ: 9.4–11.7) to 11.7 (IIQ: 10.6–12.4).

### Absence of artemisinin resistance markers.

None of the analyzed samples exhibited any WHO-validated or candidate *pfk13* mutations associated with artemisinin resistance. The only *pfk13* mutation identified was A578S, found in Matanga and Vohitromby at prevalence rates of 1% and 0.8%, respectively. This mutation has previously been reported throughout sub-Saharan Africa and is not thought to be associated with changes in artemisinin susceptibility.

## DISCUSSION

The study results have revealed ACT efficacy rates of 100% in the ASAQ arm and 99.1% in the AL arm for treating uncomplicated *P. falciparum* malaria. There was no evidence of treatment failure clustering in any geographical area, and response rates were equivalent in children under 5 years of age and older children. Mild and moderate adverse events were reported in 13.3% of patients. No mutations associated with artemisinin resistance were observed.

The treatment responses reported in the current study are consistent with those of previous studies conducted in Madagascar.^2^^–^^4^^,^^24^^,^^25^ Both ASAQ and AL exceeded the 90% threshold recommended by the WHO for considering a change in first-line antimalarial drugs.^10^ In agreement with the previous studies on ASAQ and AL mentioned above, AL and ASAQ tested in the present study induced rapid parasitic clearance within 3 days and a 50% decrease in gametocytemia over a period of 2 weeks to reach complete gametocyte elimination in most patients by the end of the study (Day 28).

No *pfk13* mutations associated with artemisinin resistance or artemisinin partial resistance were observed, suggesting continued susceptibility of circulating parasites to artemisinin. Parasitological outcome data may explain this result, as by Day 7, all children were aparasitemic. These results are consistent with those of a recent study conducted in 2018.^3^ However, it is essential to consider that human mobility or in situ selection may explain the emergence of drug-resistant malaria. In the past, the spread of *P. falciparum pfcrt* mutations from Southeast Asia to Africa was demonstrated.^26^ Additionally, the identification of parasites harboring *pfk13* mutations, along with associated delayed clearance after treatment with ACTs in eastern Africa, highlights the importance of continued monitoring for *pfk13* mutations in African therapeutic efficacy study.^7^^–^^9^^,^^27^^,^^28^

## CONCLUSION

The study results reveal that the fixed-dose combinations of ASAQ and AL are safe and efficacious for treating uncomplicated *P. falciparum* malaria in the southeastern and northwestern regions of Madagascar. These ACTs could be used for combating malaria outbreaks that often occur on the east coast of Madagascar.
